# Spiral phyllotaxis underlies constrained variation in *Anemone* (Ranunculaceae) tepal arrangement

**DOI:** 10.1007/s10265-018-1025-x

**Published:** 2018-03-27

**Authors:** Miho S. Kitazawa, Koichi Fujimoto

**Affiliations:** 1 Center for Education in Liberal Arts and Sciences, Osaka University, Toyonaka, Osaka Japan; 2 Department of Biological Sciences, Graduate School of Science, Osaka University, Toyonaka, Osaka Japan

**Keywords:** Floral organ, Variation, Floral development, Mathematical model, Phyllotaxis, Ranunculaceae

## Abstract

Stabilization and variation of floral structures are indispensable for plant reproduction and evolution; however, the developmental mechanism regulating their structural robustness is largely unknown. To investigate this mechanism, we examined positional arrangement (aestivation) of excessively produced perianth organs (tepals) of six- and seven-tepaled (lobed) flowers in six *Anemone* species (Ranunculaceae). We found that the tepal arrangement that occurred in nature varied intraspecifically between spiral and whorled arrangements. Moreover, among the studied species, variation was commonly limited to three types, including whorls, despite five geometrically possible arrangements in six-tepaled flowers and two types among six possibilities in seven-tepaled flowers. A spiral arrangement, on the other hand, was unique to five-tepaled flowers. A spiral phyllotaxis model with stochasticity on initiating excessive primordia accounted for these limited variations in arrangement in cases when the divergence angle between preexisting primordia was less than 144°. Moreover, interspecific differences in the frequency of the observed arrangements were explained by the change of model parameters that represent meristematic growth and differential organ growth. These findings suggest that the phyllotaxis parameters are responsible for not only intraspecific stability but interspecific difference of floral structure. Decreasing arrangements from six-tepaled to seven-tepaled *Anemone* flowers demonstrate that the stabilization occurs as development proceeds to increase the component (organ) number, in contrast from the intuition that the variation will be larger due to increasing number of possible states (arrangements).

**Electronic supplementary material:**

The online version of this article (10.1007/s10265-018-1025-x) contains supplementary material, which is available to authorized users.

## Introduction

**Fig. 1:**
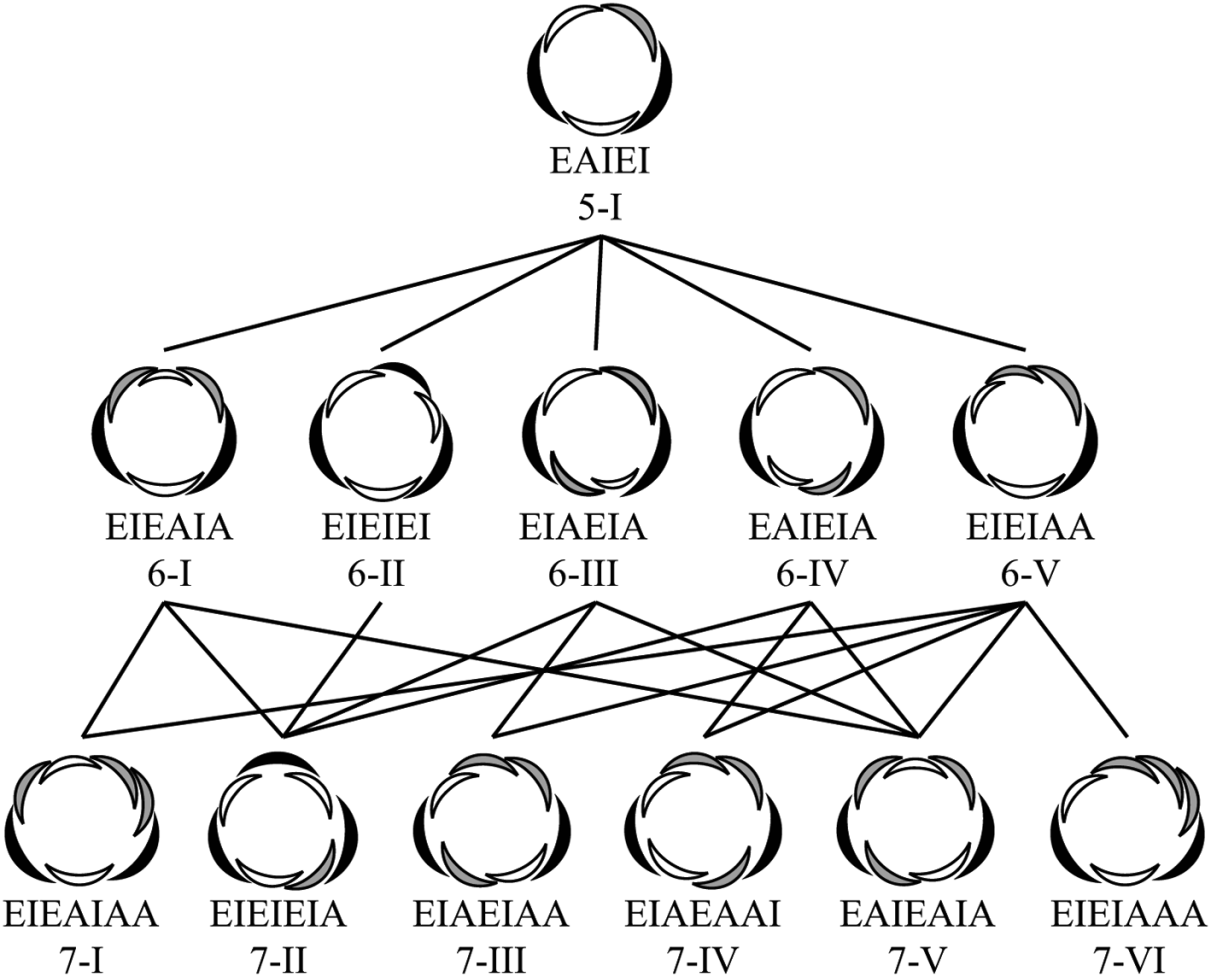
Possible arrangements for six- and seven-tepal flowers (middle and bottom rows, respectively) starting from quincuncial arrangements (top). E, I and A below floral diagrams denote external, internal, and alternating arrangement of each tepal, respectively. The sequence of these letters indicates the positional relationship of tepals in a flower

Diversification of number of floral parts (sepals and petals) is one of the major problems of floral evolution (Ronse De Craene 2016). From the ancestral state, plants evolved into a morphology with stable and clade-specific numbers of floral parts. The basic number is three in monocots, which usually have two cycles of trimerous whorls in a flower, yielding a tepal number of six (floral diagram at the left end of middle row in Fig. 1), and in Magnoliids. Alternately, the basic number is four or five in eudicots. The evolutionary course and even the ancestral state are largely unclear for these plants. One way to increase our understanding of the evolutionary change of floral morphology is to clarify the developmental mechanism that has caused such differences between clades.

The arrangement of floral parts plays a central role in the precise determination of the number of floral parts. The two major types of floral organ arrangement are spiral and whorled arrangements. Spiral initiation is often found in various clades in angiosperms (Endress and Doyle 2007). In some clades, the aestivation, the overlapping arrangement of petals or sepals is quincuncial (top row in Fig. 1; Bentley 1870). Quincuncial arrangement reflects the spiral initiation in at least some clades, such as Rosaceae and Ranunculaceae (Foster et al. 2003; Ren et al. 2010; De Craene 2010). Whorled arrangement, however, is more likely to stabilize floral part numbers than spiral arrangements, since the slight fluctuation in organ-fate determinants observed in intermediate organ morphology (e.g., between tepals and stamens; Gonçalves et al. 2013; Jabbour et al. 2015) readily affects the number in spiral arrangements (Kitazawa and Fujimoto 2014; Wang et al. 2015). Whether the spiral arrangement or the whorled arrangement of floral organs is ancestral remains controversial (Endress and Doyle 2015). Spiral initiation of floral organs is sometimes considered as the ancestral state based on the morphology of basal angiosperms (Soltis et al. 2000). On the other hand, recent estimations of the ancestral state indicated that the trimerous whorled arrangement is the ancestral state of the flowers (Sauquet et al. 2017); however, no strong evidence is available to support either of these ideas.

Flowers in the family Ranunculaceae have been a focus of studies to elucidate the developmental basis of diversity of numbers of floral organ parts (Damerval and Becker 2017; Gonçalves et al. 2013; Wang et al. 2015). The variety of numbers (2, 3, 5, and their multiples) are observed among species and within a species; therefore, these flowers offer a good system to address the question of how the floral organ number changes.

Flowers of *Anemone*, a genus in the family Ranunculaceae, consist of multiple pistils, stamens, and surrounding perianth organs (tepals), which are usually white, pink, or purple but not green as sepals of core eudicots. As in other genera of Ranunculaceae, *Anemone* flowers show inter- and intra-specific diversity in the number of floral parts (Schöffel 1932; Yule 1902). In fact, this genus includes species, such as *A. nemorosa* and *A. hepatica* (*Hepatica nobilis*), with two cycles of trimerous whorls as well as spiral flowers, including *A. nikoensis, A. flaccida*, and *A. scabiosa* (*A*. × *hybrida*; Japanese anemone), with a typical tepal number of five (Kitazawa and Fujimoto 2016a). These species are not separately clustered but rather scattered in the phylogenetic tree (Hoot et al. 2012). Furthermore, trimerous-like arrangements can be stochastically found even in the species with pentamerous spiral flowers (Kitazawa and Fujimoto 2016b; Ren et al. 2010; Schöffel 1932). The development of some *Anemone* flowers indicates a transient pattern between spiral and trimerous whorls: first three tepal primordia arise sequentially, and then three tepal primordia initiate at once (Ren et al. 2010). Therefore, the *Anemone* flowers with six tepals can be considered a primitive form of the trimerous whorl, suggesting that whorled arrangement and spiral initiation is closely related in developmental regulation.

Previously, we studied the variation in tepal arrangements of flowers with six tepals in wild populations of three *Anemone* species and found that variation was limited to three aestivation types, including trimerous double whorl type (floral diagram at the left end of middle row in Fig. 1), despite the option of five geometrically possible types (Kitazawa and Fujimoto 2016b). To examine this limited variation associated with the increasing number of tepals, we analyzed the position of the seventh as well as the sixth tepals of *Anemone* in both wild populations and a floral phyllotaxis model. We examined the possible consequences of tepal appearance and resultant arrangements from each position of the sixth tepal, assuming that the seventh tepal appears after the sixth tepal and any tepal does not overlap with others (Fig. 1). By comparing actual arrangements with the possible options, we demonstrate that the arrangement stabilizes as the number of components (tepals) increases.

## Materials and methods

### Positional arrangement of perianth organs

The arrangement of tepals was examined for flowers with five, six, and seven tepals, whereas the arrangement was not determined for flowers with more than seven tepals. Based on previous studies (Cunnell 1958; Kitazawa and Fujimoto 2016b; Morgan 1874; Schoute 1935), we surmised the aestivation by positional arrangements of tepals in mature flowers by identifying the arrangement of each organ with neighboring organs as either external, internal, or alternating (Fig. 1). For example, type 5-I represents quincuncial, whereas type 6-II represents three internal and three external tepals, exemplifying trimerous double whorls. For simplicity, reflected and rotated arrangements were not distinguished, and therefore, the position of the flower with respect to the main axis and the direction of the spiral was ignored. For five-tepaled flowers, there are four geometrically possible aestivations (Cunnell 1958). For six-tepaled flowers derived from quincuncial, there are five aestivation types (Fig. 1 middle row; Kitazawa and Fujimoto 2016b). Regarding flowers with seven tepals, since the arrangement type 6-II is radially symmetric, the resulting arrangement converge onto only the type 7-II (Fig. 1 lower). When the arrangements are type 6-I or 6-IV, the arrangement at the sixth tepal is bilateral. Therefore, three possible arrangements exist for seven-tepaled flowers: types 7-I, 7-II, and 7-V for type 6-I and types 7-II, 7-IV, and 7-V for type 6-IV. Type 6-III is a biradially symmetric arrangement, and therefore, this arrangement can generate three arrangements of seven-tepaled flowers: types 7-II, 7-III, and 7-V. The non-symmetric arrangement type 6-V has five possibilities: types 7-I, 7-III, 7-IV, 7-V, and 7-VI.

### Plant samples

We measured the positional arrangements of tepals in wild populations of *A. nikoensis* (Shiga, Osaka, Hyogo and Okayama prefectures), *A. flaccida* (Hokkaido, Shiga, Hyogo and Okayama pref.), *A. soyensis* (Hokkaido pref.), *A. hepatica (Hepatica nobilis;* Shiga and Okayama pref.), *Pulsatilla cernua* (Hyogo pref.), and three forms of *Anemone* × *hybrida* (Japanese anemone; we previously called *A. scabiosa* in Kitazawa and Fujimoto 2016a, b; Hokkaido, Tokyo, Mie, Shiga, Kyoto, Nara, Osaka and Hyogo pref.) in Japan. The frequency of each arrangement was measured as the sum of the multiple populations within the species (Table S1). Although there are several forms of *A*. × *hybrida*, we were unable to identify the forms at many of our observation sites. Therefore, we used tepal color as the primary feature to define the forms. Based on tepal color, we could clearly distinguish the three groups: deep pink, pale pink, and white. Populations with deep pink tepals are further classified as types with broad, obovate tepals or with thin, numerous tepals (more than 10). While the latter was excluded from our study, we examined the pale-pink, white, and deep-pink broad tepal groups. *Pulsatilla* is nested in *Anemone* in molecular phylogenetic studies (Hoot et al. 2012; Jiang et al. 2017). We counted only the fresh flowers, because tepals became narrower at their basal parts and positional overlaps were lost as days went on after blooming.

### Mathematical model settings

**Fig. 2:**
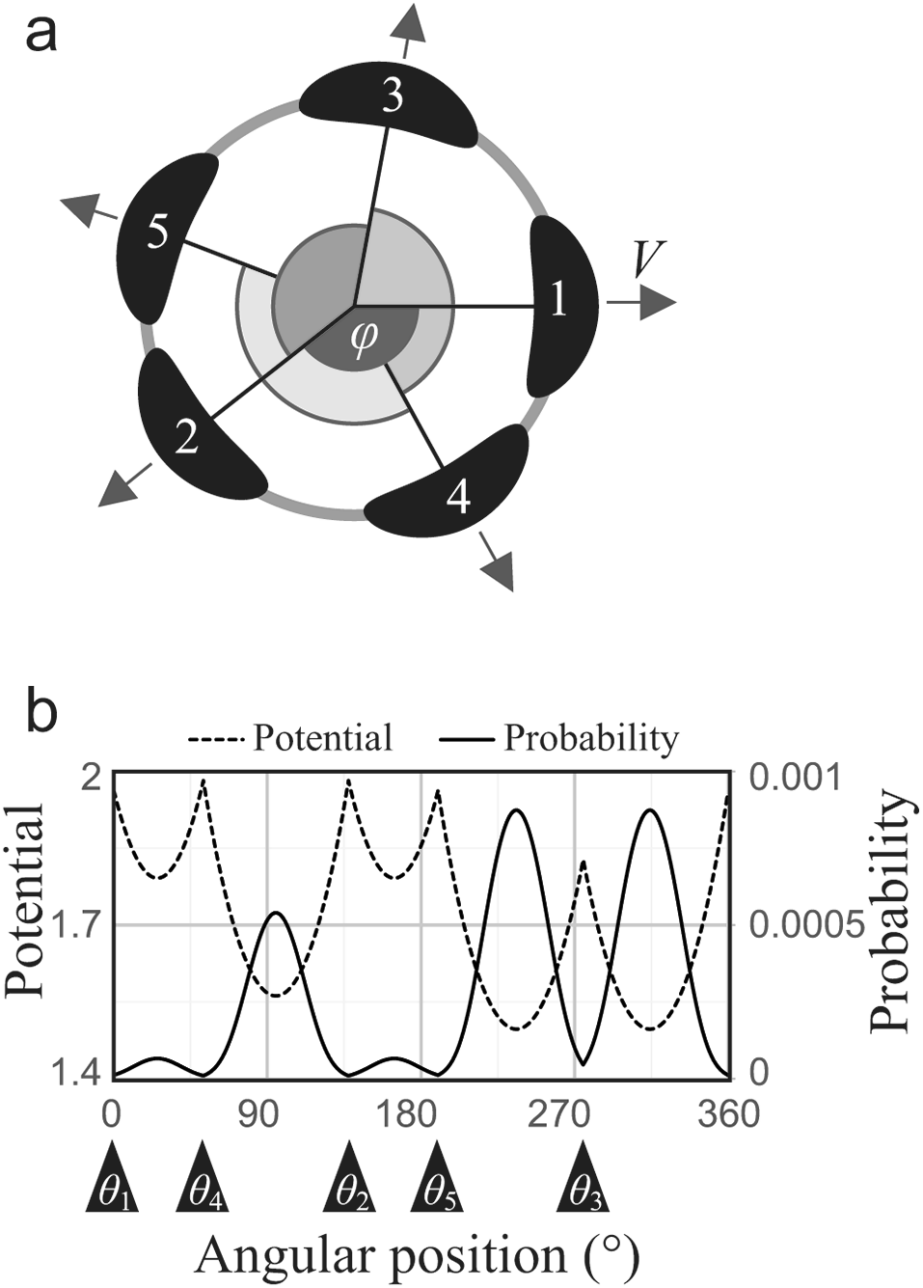
Model settings. The first five primordia appear sequentially with fixed divergence angle φ at the edge of the floral meristem (**a**). The positions of the fifth and onward primordia are determined by the probability (Eq. 3) calculated from the inhibitory energy (Eq. 2) of the existing primordia (**b**). The potential and probability are calculated discretely with an interval of 0.1°. R_0_ = λ = 10, *φ* = 137.5°, *VT* = α = 0, and *b* = 10

To study the stochasticity in positional arrangement of the sixth and seventh tepals, we employed the inhibitory field model as described by Douady and Couder (1996). We assumed that the primordia sequentially appear one by one at the periphery of the circular meristem with a radius of R_0_, following the observation of early development of *Anemone* (Ren et al. 2010). Radial position of primordium *j* at the initiation of primordium *i* was set as

1 $${{\text{r}}_j}={\text{ }}{{\text{R}}_0}+{\text{ }}\left( {i - j} \right)VT,$$

where *VT* is a centrifugal displacement of each primordium reflecting meristem growth with a velocity *V* during the time interval *T* of initiation of two successive primordia (Fig. 2a). To simplify the situation, the divergence angle *φ* between the successive *i*-th and *i* + 1-th primordia were fixed (1 ≦ *i* ≦ 4). The angle must be satisfied with 120° < *φ* < 180° to reproduce the quincuncial aestivation of flowers with five tepals (Fig. 1, top). The angular positions of sixth and later primordia *θ*_*i*_ (*i* ≧ 6) were determined stochastically based on the inhibitory effect from existing primordia (Fig. 2b, dashed line). Denoting an index of the current primordium as *i* (*i* ≧ 6) and those of existing primordia as *j* (1 ≦ *j* < *i*), the potential energy was formulated based on a previous study of floral phyllotaxis (Kitazawa and Fujimoto 2015, 2016b) as

2 $${E_{\theta ,i}}={\text{ }}\sum\nolimits_{j} {{\text{exp}}\left( {\alpha \left( {i - j} \right)} \right){\text{exp}}\left( { - {d_{ij}}/\lambda } \right),}$$

where *d*_*ij*_ and λ are the distance between primordia *i* and *j* and a parameter representing effective distance of inhibition, respectively. In addition, α represents the difference of the inhibitory effect due to growth progression of pre-existing organs, whose positive/negative sign is identical to that from an earlier study (Kitazawa and Fujimoto 2015) but opposite of that from another one (Kitazawa and Fujimoto 2016b). The condition *VT* = 0 corresponds to the situation described in the previous study (Kitazawa and Fujimoto 2016b), while for simplicity, the present model does not incorporate the angular displacement of primordia, which was introduced after primordia initiation in an earlier study (Kitazawa and Fujimoto 2015). The α accounts for the change of the direction of auxin flux toward the inner tissue of primordia and/or primordial boundary establishment and the increase of primordial volume. Since lower energy indicates a higher potential (probability) to place a new primordium, the probability *p*_*θ,i*_ is given by a monotonically decreasing sigmoidal function of the energy,

3 $${p_{\theta ,i}}={C_0}/\left( {{\text{1}}+{\text{exp}}\left( {b\left( {{E_{\theta ,i}} - \left( {{\mu _E} - {\text{2}}{\sigma _E}} \right)} \right)} \right)} \right),$$

where *b* is a parameter that indicates the steepness of the sigmoidal function, and *µ*_*E*_ and *σ*_*E*_ are the average and standard deviation of energy $${\mu _E} \equiv \int_{0}^{{360}} {{E_{\theta ,i}}d\theta /{\text{36}}0}$$ and $${\sigma _E} \equiv {\left( {\int_{0}^{{360}} {{{({E_{\theta ,i}} - {\mu _E})}^{\text{2}}}d\theta /{\text{36}}0} } \right)^{{\raise0.5ex\hbox{$\scriptstyle 1$}\kern-0.1em/\kern-0.15em\lower0.25ex\hbox{$\scriptstyle 2$}}}}$$, respectively. *C*_*0*_ is a normalization constant that satisfies the function $$\int_{0}^{{360}} {{p_{\theta ,i}}d\theta /{\text{36}}0={\text{1}}}$$. Using this probability, we numerically obtained the stochastic arrangement of the primordia (Mirabet et al. 2012; Refahi et al. 2016; Fig. 2b).

## Results

### Species-dependent appearance of spiral pentamery and whorled trimery

**Fig. 3:**
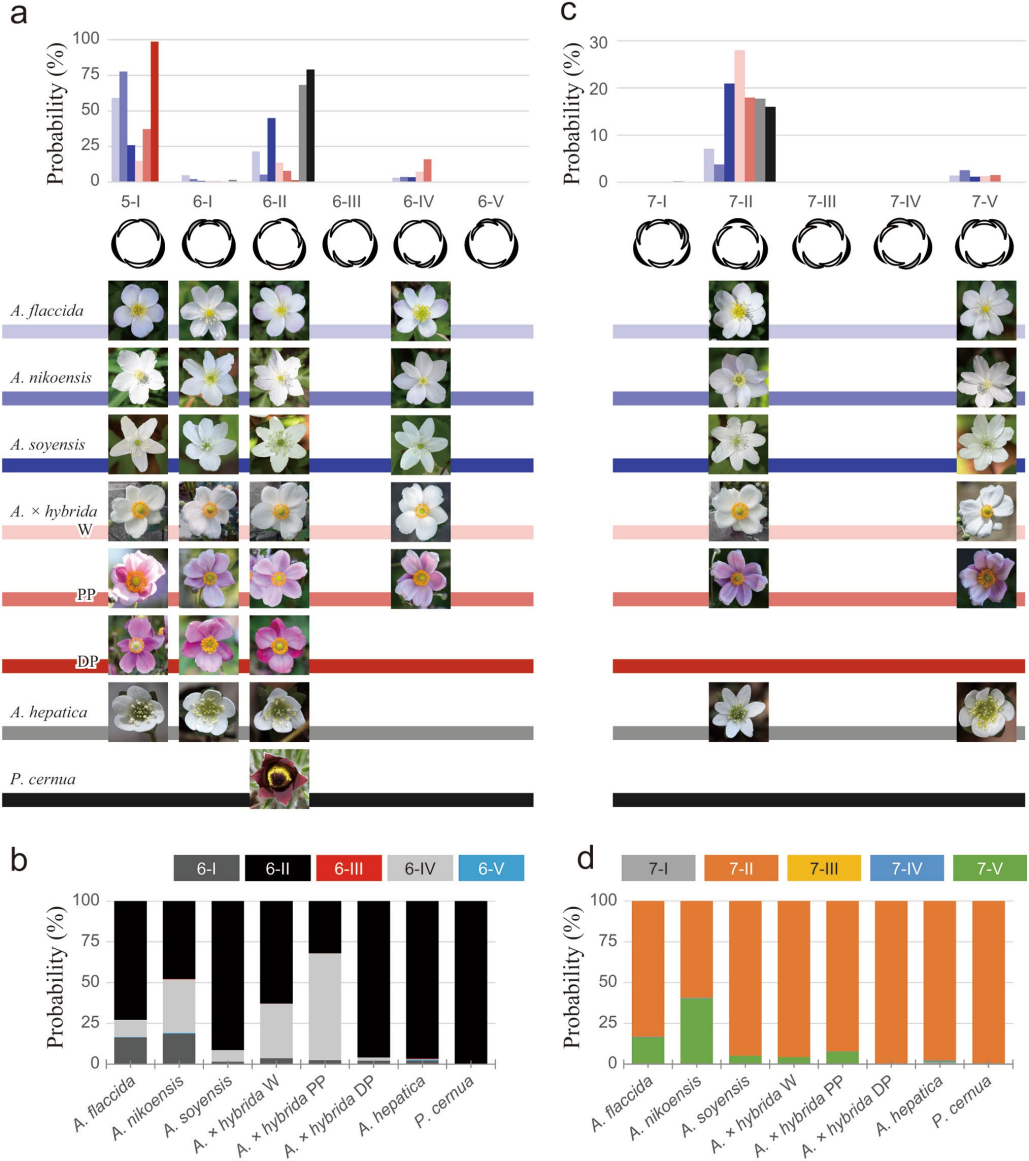
Probability of perianth arrangements of flowers with five or six tepals (**a, b**) and those with seven tepals (**c, d**) measured for six *Anemone* species. The chart indicates the frequency of each arrangement normalized by all record counts of the species regardless of the tepal numbers (**a, c**), whereas the stacked bar chart shows the frequency of the arrangements normalized by the number of six-tepaled (**b**) and seven-tepaled (**d**) flowers. Photographs under charts (**a, b**) are representatives of each arrangement of each species and form, whose brightness is modified to clearly show the arrangements

For five-tepaled flowers, almost all flowers underwent a unique aestivation type (*n* = 20,286 among 20,287 five-tepaled *Anemone* flowers). That is, the flowers were quincuncial type 5-I (Fig. 3a), which is consistent with spiral initiation of organ primordia during floral development (Ren et al. 2010). For flowers with six tepals, we examined the frequency of aestivation types. The most frequent aestivation type was type 6-II, except for the *A*. × *hybrida* pale pink form, which exhibited the type 6-IV arrangement most frequently (Fig. 3a, b), as reported previously (Kitazawa and Fujimoto 2016b). Notably, whether pentamerous, quincuncial (type 5-I), or trimerous double whorls (type 6-II) was more frequent was species-dependent: quincuncial in *A. nikoensis, A. flaccida*, and *A*. × *hybrida* deep pink and pale pink and trimerous double whorls in *A. hepatica, A. soyensis*, and *Pulsatilla cernua* (Fig. 3a). The frequencies of quincuncial and type 6-II were nearly equivalent in *A*. × *hybrida* white. The normalized frequency of pentamerous quincuncial further varied among the four observed plant types: *A*. × *hybrida* deep pink (99%), *A. nikoensis* (78%), *A. flaccida* (59%), and *A*. × *hybrida* pale pink (37%).

### Limited aestivation in six-tepaled flowers

In species showing higher frequency of type 6-II than quincuncial (*A. soyensis, A. hepatica*, and *P. cernua*), the frequency of type 6-II normalized to that of six-tepaled flowers was more than 90%, indicating outstanding robustness of type 6-II (Fig. 3a). On the other hand, in those showing equal or higher frequency of quincuncial (type 5-I) than type 6-II (*A. flaccida, A. nikoensis*, and *A*. × *hybrida*), types 6-I and 6-IV also appeared at comparable frequency to type 6-II, whereas the other two aestivation types (types 6-III and 6-V) were rare as reported previously (Kitazawa and Fujimoto 2016b; Fig. 3a, b). We confirmed the constrained appearance to three aestivation types of six-tepaled flowers by measuring a four times larger sample size (~ 9 × 10^3^ six-tepaled flowers in all populations of observed species; Fig. 1) than in the previous report. These three types (types 6-I, 6-II, and 6-IV) dominated 99.3% of the six tepaled-flowers in all the observed species and forms (Fig. 3b). The frequency rank of the three types was type 6-II, type 6-IV, and type 6-I in ascending order for *A. nikoensis* and *A*. × *hybrida* white, whereas this order was types 6-II, 6-I, and 6-IV for *A. flaccida* and types 6-IV, 6-II, and 6-I for *A*. × *hybrida* pale pink. Therefore, six-tepaled flowers of pentamerous species (*A. flaccida, A. nikoensis*, and *A*. × *hybrida*) exhibited limited variation of three aestivation types in a species- and form-dependent manner, while five-tepaled flowers of trimerous species (*A. soyensis, A. hepatica*, and *P. cernua*) were uniquely quincuncial.

### Limited aestivation in seven-tepaled flowers

As a consequence of limited aestivation of flowers with six tepals, we analyzed the aestivation of seven-tepaled flowers that co-exist with the five- and six-tepaled flowers in wild populations (Fig. 3c). Geometrically, there are ten possible aestivation types for seven-tepaled flowers (Fig. 1 lower row and Fig. S1). Even when we assume that the flowers with seven tepals are generated by adding two tepals to the inside of the quincuncial aestivation of five-tepaled flowers, six possible aestivation types remain (lower row in Fig. 1). Intriguingly, we found that two aestivation types (types 7-II and 7-V) appeared much more frequently than the other four types, dominating 98.3% of the seven-tepaled flowers in all observed species and forms (Fig. 3d). In addition, the frequency ratio of type 7-II to type 7-V was greater than 5, indicating outstanding robustness of type 7-II in all species and forms, except for *A. nikoensis*, where this frequency ratio was approximately 1.5, indicating similar robustness of the two aestivation types.

### A model for spiral phyllotaxis with stochasticity reproduced limited variation of *Anemone* tepal arrangements

**Fig. 4:**
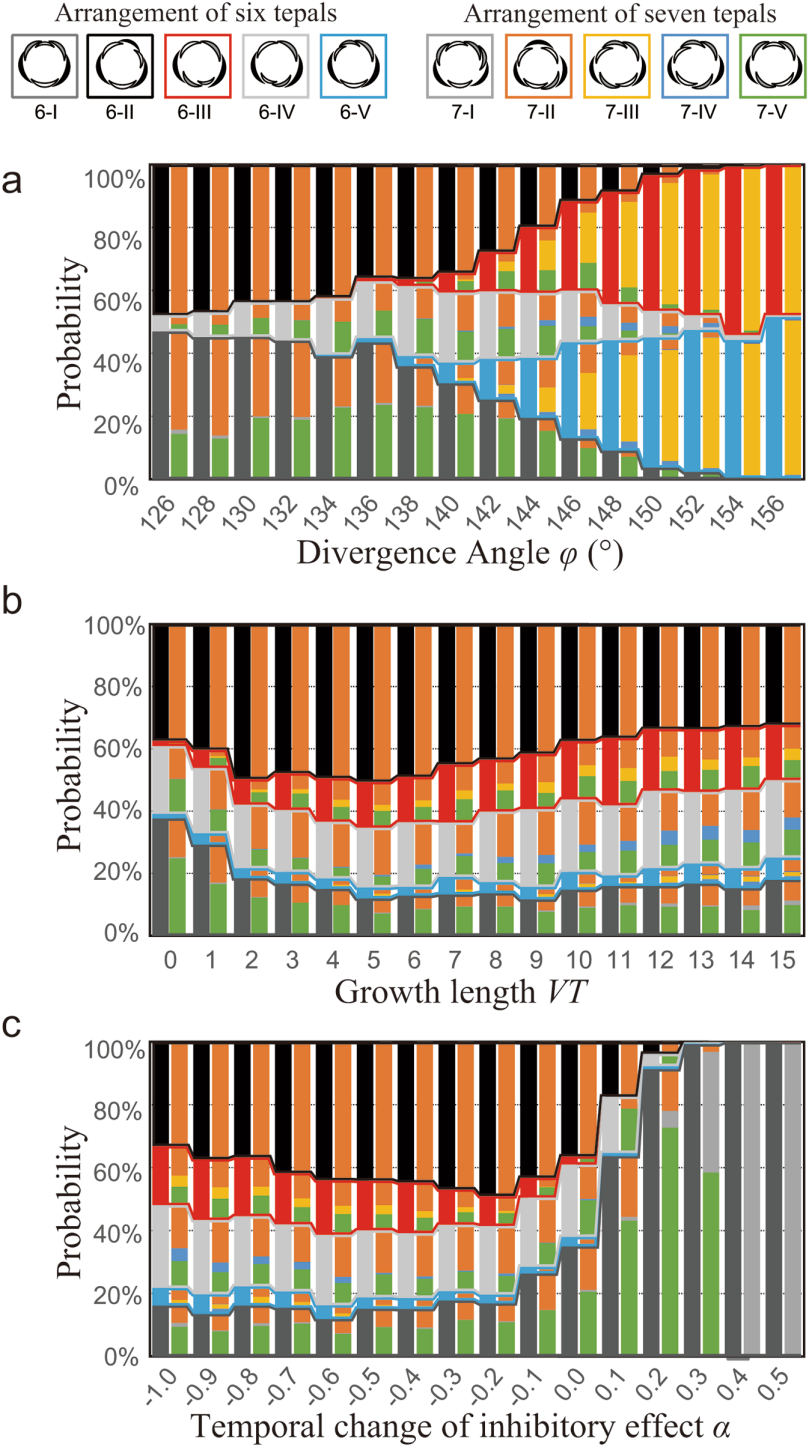
Probability of perianth arrangements in spiral phyllotaxis model simulations. A pair of stacked bars indicating the arrangements at the appearance of sixth (left bar) and seventh tepal (right bar) is shown for each parameter value. The dependency on divergence angle φ (**a**), the growth length *VT* (**b**), and α (**c**) are shown. R_0_ = λ = 10, *b* = 10, *VT* = α = 0, and *φ* = 137.5° if the values are not specified

To understand the developmental mechanisms of the limited positions of sixth and seventh tepals, we first examined the arrangements of the six tepals at the time when the sixth tepal appeared after the quincuncial arrangement in a phyllotaxis model using computational analysis (Fig. 2). The simplest case includes a divergence angle *φ* = 144° and no growth (*VT* = 0; Fig. 4a), since the first five tepals are placed in the regular pentagon. The computational simulation was consistent with intuitive expectation. That is, the five possible arrangements of the six tepals appeared with equal probability (*φ* = 144° in Fig. 4a). The dependency on *φ* without growth was also consistent with a previous theoretical study based on the energy landscape (Eq. 2; Kitazawa and Fujimoto 2016b). Three of the arrangements (types 6-I, 6-II, and 6-IV) observed in *Anemone* (Fig. 3a, b) appeared when *φ* < 144°, whereas the other two (types 6-III and 6-V) appeared when *φ* > 144° (Fig. 4a). Next, we checked the position of the seventh tepal for each arrangement and found that the seventh tepal position was also limited. Two arrangements (types 7-II and 7-V) that were dominant in *Anemone* (Fig. 3c, d), occupied nearly all the arrangements at *φ* < 144°, despite the six possible arrangements of seven tepals initiating from the quincuncial arrangement (Fig. 1, bottom row). At *φ* > 144°, the two arrangements decrease in frequency, and type 7-III, which was virtually not observed for *Anemone* (Fig. 3c, d), becomes dominant, increasing in frequency as *φ* gets larger (Fig. 4a). Type 7-IV (rank 3 except for *A. hepatica*) appeared and increased to a frequency of up to 6% when *φ* was approximately 144° (maximum value at *φ* = 146°) but decreased as *φ* was further increased. Type 7-I (rank 3 in *A. hepatica* and rank 4 in *A. nikoensis*) appeared at a very low frequency (maximum 1.4% at *φ* = 126°), decreased as *φ* neared 144, and then increased again (0.8% when *φ* = 156°). Type 7-VI (not observed in *Anemone*) was consistently nearly absent (0.4% at *φ* = 154° was the maximum) throughout the parameter range examined. Therefore, we conclude that the dominant frequency of types 7-II and 7-V for seven-tepaled flowers as well as types 6-I, 6-II, and 6-IV for six-tepaled flowers is a natural consequence of spiral phyllotaxis with *φ* < 144°.

Next, we examined the dependency of centrifugal displacement due to growth *VT* (Fig. 4b). For the sixth tepal arrangements, as *VT* gets larger, the fraction of type 6-II increased until *VT* = 4 and then decreased as *VT* increased further. At the same time, the fraction of type 6-III increased, while the fraction of type 6-IV did not change and the fraction of type 6-I decreased to 10–20% at *VT* = 1. The position of the seventh tepal showed higher variation than for the case when *VT* = 0. For example, the position of the seventh tepal subsequent to the arrangement type 6-IV converged to two arrangements (types 7-II and 7-V), but another arrangement (type 7-IV) also appeared when *VT* was larger than 4 (Fig. 4b). Overall, as *VT* increased, a higher degree of variation in the arrangements with seven-tepaled flowers was observed. Therefore, the arrangements with seven tepals were limited to two dominant types (types 7-II and 7-V) at small *VT* but were more varied as *VT* increased.

Finally, we examined the dependency of arrangements on another parameter, α representing thedifference of inhibitory effect due to growth progression on pre-existing organs (Fig. 4c; see “Materials and Methods” for definition of α). The limitation to three types (types 6-I, 6-II, and 6-IV) in *Anemone* flowers with six tepals and two types (types 7-II and 7-V) in those with seven tepals was reproduced for 0.05 ≲ α ≲ 0.1. As α represents a bias of inhibitory effect according to the tepal indices (Eq. 2), negative α had an effect similar to that of *VT*: For the flowers with six tepals, as α decreased, the fraction of type 6-III increased, similar to the case when *VT* increased, while rank of types 6-II, 6-IV, and 6-I in six-tepaled arrangements remained at 1, 2, and 3, respectively (Fig. 4c), The arrangements with seven tepals were more variable when α was small, as in the case when *VT* was larger. On the other hand, as α increased, the fraction of type 6-I increased, changing its rank among arrangements of six tepals from 3 to 2 and 1 (see α > − 0.2 in Fig. 4c), consistent with a previous study (Kitazawa and Fujimoto 2016b). In the arrangement with the seventh tepal, type 7-V was most dominant above a positive threshold value of α ~ 0.1, whereas the fraction of type 7-I subsequently increased to be the most dominant arrangement as α further increased. Of the parameter range examined, a positive α is the only condition for which the arrangement type 7-I appeared. In summary, a larger *VT* and smaller negative α cause greater variation in arrangements; therefore, the arrangements are not limited to a small number of types. A larger space between primordia caused by larger *VT* and a decrease of inhibition due to negative α both decrease the roughness of the energy landscape, resulting in gently sloping probability density over the edge of the floral meristem. Hence, limitation to small number of arrangement types implies that the development proceeds in compact packing of organ primordia.

## Discussion

### Phyllotaxis of seven-tepaled flowers is biased by those of six-tepaled flowers

**Fig. 5:**
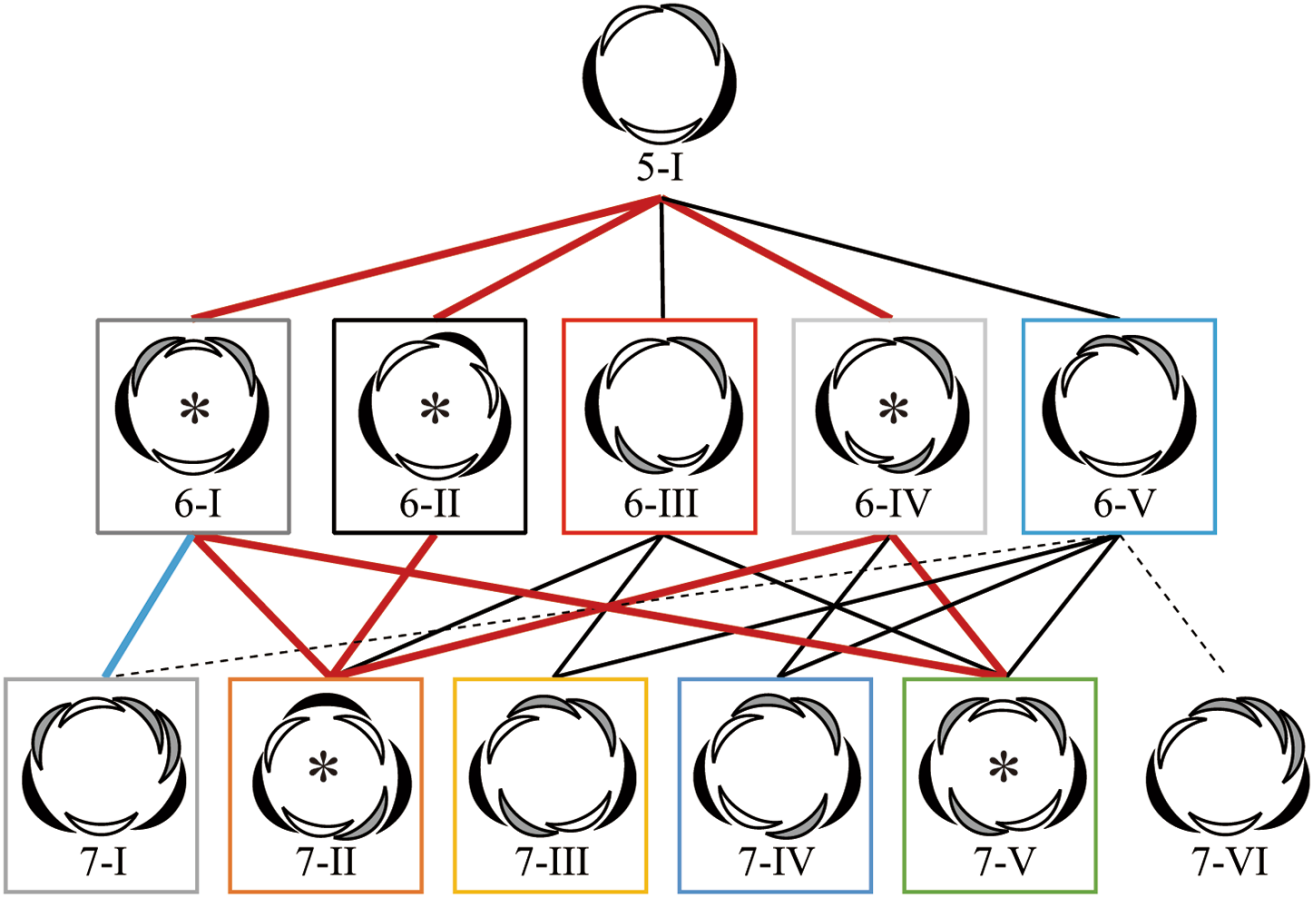
Summary of the limited arrangement of five- (top), six- (middle), and seven- (bottom) tepaled flowers as they consistently appeared in *Anemone* and spiral phyllotaxis model (asterisks). Frame colors correspond to the simulation results in Fig. 4. The arrangement that was not found in the simulations is not boxed. Red paths indicate the arrangements that appear at φ < 144° and *VT* = α = 0, whereas a blue path indicates the arrangement appeared only at *α* > 0. The arrangements appeared at any other parameters are indicated by black paths, whereas those hardly appeared in simulations are indicated by dashed paths

Using the results of the numerical simulation, we can depict the possible pathways of development (Fig. 5). For example, type 7-VI can geometrically appear by adding one tepal inside the type 6-V (Fig. 5, dashed line), but this arrangement hardly appeared in simulations. Some paths appeared only for limited range of parameters. For example, the path from type 6-I to type 7-I only occurred when α was positive (Fig. 5, blue solid line). As we observed three arrangements of six tepals [types 6-I, 6-II, and 6-IV (Fig. 3a, b)], the parameter *φ* is likely to be less than 144 (Fig. 4a) in the field, and α is not a large positive number (Fig. 4c). Within this parameter region, the paths towards type 7-II and type 7-IV were the most frequent in the simulation (Fig. 5, red lines); therefore, the frequent appearance of these two arrangements in association with the high frequency of type 6-I, type 6-II, and type 6-IV is consistent with the spiral phyllotaxis. Thus, we expect that if a population has a high frequency of type 6-III, then the population will exhibit a frequent appearance of type 7-III (Fig. 5, black lines).

### Possible developmental origins of the constrained variations

When do these constrained arrangements emerge during floral development? In pentamerous *Anemone* species, tepal primordia initiate sequentially in a spiral order (e.g. *A. taipaiensis*, Fig. 1c and 7 in Ren et al. 2010), resulting in a quincuncial arrangement (type 5-I). On the other hand, in trimerous species (e.g. *A. chinensis*, Fig. 1f and 13 in Ren et al. 2010), three primordia emerge at the same time or in rapid succession, therefore it can be regarded as a trimerous whorl. Interestingly, in *A. tomentosa* with five or six tepals, tepal primordia initiate in a spiral order in a five-tepaled flower, whereas the primordia locate at the positions of trimerous double whorls (type 6-II) in a six-tepaled flower (Fig. 1d and 10 in Ren et al. 2010). Therefore, such stochastic appearance of trimerous whorls can occur by changing position and/or timing of primordia initiation. On the other hand, to our knowledge, the other arrangements presented here in mature flowers (types 6-I, 6-IV, 7-II, and 7-V) have not been reported at floral organ primordia initiation stage in angiosperms. These arrangements would emerge at an early stage in floral development, or through the post-meristematic process, where internode length between successive primordia are dynamically regulated (Kitazawa and Fujimoto 2015; Peaucelle et al. 2007). The developmental process of these arrangement types is an interesting future problem of experimental botany to estimate the importance of post-meristematic process on stabilizing floral structure.

### Consistency of a stochastic spiral model with *Anemone*

Both in field observations (Fig. 3) and numerical simulations (Fig. 4), we identified a clear bias of arrangement appearance in both six- and seven-tepaled flowers. In *A. nikoensis* and *A. flaccida*, type 6-I had a relatively high frequency in the flowers with six tepals (18.7 and 16.3%, respectively), whereas this frequency was low (less than 4%) in other species (Fig. 3b). These two species also showed higher frequencies of type 7-V among flowers with seven tepals compared with other species (39.8 and 16.4%, respectively; Fig. 3d). This increased frequency of type 6-I in association with the increase of type 6-IV was also found in the numerical simulations with an α = − 0.1 (Fig. 4c). Therefore, we expect that with a small positive α, the older primordia have equal or slightly greater inhibitory effects on the initiation of a new primordium than newer primordia in these two species, whereas this effect appears to be smaller in other species. On the other hand, the present simulations did not account for a frequency of more than 90% for type 6-II in species *A. soyensis, A. hepatica*, and *P. cernua* (Fig. 3b), where the double-whorled arrangement was more frequent than quincuncial (Fig. 3a), suggesting that any other developmental properties that were not incorporated into the present model regulate the stabilization of trimerous double whorls in *Anemone*. Exploring these mechanisms is an exciting future direction of floral phyllotaxis modelling.

Although the number of possible arrangements increases as the number of tepals increases from six to seven, the number of actual arrangements decreased. Intuitively, the increase of components number is likely to correlate with an increase in variation. Our results suggest, however, that the process of development and increased component number can stabilize the overall structure. Our results also suggest a direct path from quincuncial arrangement to the arrangement of two cycles of trimerous whorls. One interesting unanswered question regarding floral evolution involves the path of development when the component number is further increased. For example, flowers with eight tepals may have an arrangement related to two cycles of tetramerous whorls. Understanding the stabilization of such an arrangement would illuminate the evolutionary pathway for precise determination and species diversity of floral part numbers.

## Conclusion

In this study, we demonstrated that the arrangements of tepals in six- and seven-tepaled *Anemone* flowers are limited to a small number of arrangement types in stark contrast with the intuitive notion that the variation would be greater due to an increasing number of possible states (arrangements). This limitation was explained by a mathematical model of spiral phyllotaxis. Our results indicate that the spiral nature underlies the stabilization process with increasing component (organ) number, yielding a canalization of organ arrangements (phyllotaxis) as development proceeds.

## Electronic supplementary material

Below is the link to the electronic supplementary material.

Supplementary material 1 (PDF 158 KB)
