# Supplementary material for: Spiral phyllotaxis underlies constrained variation in Anemone (Ranunculaceae) tepal arrangement
Source: J Plant Res. 2018 Mar 27;131(3):459–68. doi: 10.1007/s10265-018-1025-x (PMC5916976; doi:10.1007/s10265-018-1025-x)

Electric supplementary materials

Title:

Spiral phyllotaxis underlies constrained variation in *Anemone* (Ranunculaceae) tepal arrangement

Authors:

Miho S. Kitazawa and Koichi Fujimoto

Journal:

Journal of Plant Research

Corresponding author:

Miho S. Kitazawa

Center for Education in Liberal Arts and Sciences, Osaka University

Department of Biological Sciences, Graduate School of Science, Osaka University

1-16 Machikaneyama-cho, Toyonaka, Osaka, 560-0043, Japan

06-6850-8124

kitazawa@celas.osaka-u.ac.jp

Koichi Fujimoto

Department of Biological Sciences, Graduate School of Science, Osaka University

1-1 Machikaneyama-cho, Toyonaka, Osaka, 560-0043, Japan

06-6850-5822

fujimoto@bio.sci.osaka-u.ac.jp

Content:

Table S1

Fig. S1

Table S1 Absolute frequency of *Anemone* tepal arrangements in Fig. 3. The other arrangements were not observed. See Fig. 1 for the arrangement types and the use of letters E, I, and A.

Tepal number	under 5	5	5	6	6	6	6	6	7	7	7	7	7	above 7	Total number of Flowers	Number of Populations
Tepal arrangement type	unidentified	5-I	EAAAI	6-I	6-II	6-III	6-IV	6-V	7-I	7-II	7-III	7-IV	7-V	unidentified		
A. flaccida	7	3750	0	308	1371	1	198	6	0	451	0	2	89	166	6349	15
A. nikoensis	79	8526	1	229	583	3	403	5	3	413	1	3	278	455	10982	39
A. soyensis	0	67	0	2	116	0	9	0	0	54	0	0	3	7	258	2
A. ×hybrida W	0	1650	0	86	1516	1	809	2	0	3096	0	1	143	3757	11061	128
A. ×hybrida DP	0	3657	0	1	45	0	1	0	0	2	0	0	0	1	3707	12
A. ×hybrida PP	0	2630	0	43	553	1	1132	0	0	1267	0	1	107	1323	7057	72
A. hepatica	0	5	0	15	674	2	2	5	2	175	0	1	1	105	987	4
Pulsatilla cernua	0	1	0	1	1030	1	0	0	0	208	0	0	1	61	1303	2

Fig. S1 Four possible arrangements of seven tepals, which cannot be derived from the quincuncial arrangement (Fig. 1).

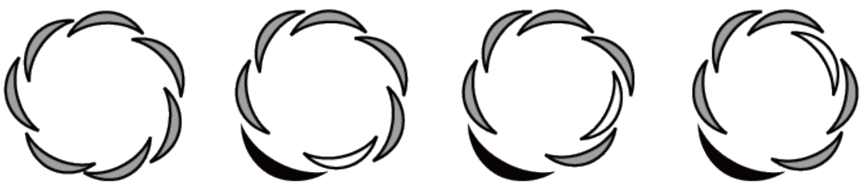

Supplement: Supplementary file 1 — Supplementary material 1 (PDF 158 KB) [file 10265_2018_1025_MOESM1_ESM.pdf]
